# Promoção de Dietas Saudáveis para o Enfrentamento de Doenças Cardiovasculares no Brasil: A Importância das Diretrizes Nutricionais e Políticas Públicas

**DOI:** 10.36660/abc.20240378

**Published:** 2024-08-02

**Authors:** Laís Ferreira Dias, Aline Martins de Carvalho

**Affiliations:** 1 Departamento de Nutrição Faculdade de Saúde Pública Universidade de São Paulo, São Paulo Brasil Departamento de Nutrição da Faculdade de Saúde Pública da Universidade de São Paulo, São Paulo – Brasil; 2 Instituto do Coração Faculdade de Medicina Universidade de São Paulo São Paulo SP Brasil Instituto do Coração (InCor), Faculdade de Medicina - Universidade de São Paulo, São Paulo, SP – Brasil

**Keywords:** Doenças Cardiovasculares, Sistema Alimentar, Prevenção de Doenças, Saúde Pública

As doenças cardiovasculares (DCV) representam um problema de saúde pública de alta magnitude no Brasil, sendo a principal causa de mortalidade no país, com destaque para a doença arterial coronariana como a principal causa de óbitos.^1^Segundo os dados do *Global Burden of Disease*, 2021, o padrão alimentar foi responsável por 12% do risco atribuído ao total de mortes por doenças crônicas não transmissíveis na população geral brasileira.^2^ Tais condições ocasionam prejuízos sistêmicos, não apenas impactando na mortalidade populacional ou reduzindo a expectativa de vida, mas também a incapacidade, gerando dependência de um indivíduo para realizar suas atividades cotidianas.^3^

Diante deste cenário, a Organização Mundial de Saúde (OMS) defende políticas públicas focadas em estratégias de saúde e atualização regular das diretrizes nacionais de alimentação e nutrição, adaptando-se às mudanças nos hábitos alimentares, nos sistemas alimentares, e nas condições de saúde da população.^4,5^

No Brasil, há diversas diretrizes nutricionais e programas governamentais direcionados à promoção de dietas saudáveis. O Programa Alimentar Brasileiro Cardioprotetor (DICABR), tem como objetivo promover uma alimentação saudável e adequada para prevenir doenças cardíacas e categoriza os alimentos em grupos identificados pelas cores da bandeira do Brasil, explorando a cultura e a regionalidade alimentar do país. A cor verde representa os alimentos que devem ser consumidos em maior quantidade, como frutas, legumes, verduras, leites e iogurte. A cor amarela, alimentos que devem ser consumidos com moderação, como pães, cereais, tubérculos e alguns tipos de doces caseiros (cocada, goiabada, doce de abóbora). A cor azul representa alimentos que devem ser consumidos em menor quantidade, sendo estes carnes, queijos, ovos, manteiga, leite condensado e creme de leite. Apesar de seu potencial, a adesão e a divulgação entre a população beneficiária foram insuficientes para alcançar os resultados esperados na prevenção de DCV.^6^

Um estudo transversal realizado com indivíduos em centros de referência cardiovascular no Nordeste do Brasil evidenciou que a população estudada está longe de cumprir as recomendações do DICABR.^7^ A dieta desses indivíduos foi pobre em fibras e micronutrientes, com alta ingestão de gorduras saturadas e baixo consumo de ácidos graxos insaturados, evidenciando sua baixa qualidade nutricional.

Um outro estudo piloto randomizado e controlado avaliou 117 pacientes ambulatoriais brasileiros com mais de 45 anos de idade com doença cardiovascular aterotrombótica. Apesar da ausência de significância estatística, o estudo ressalta o potencial do consumo de uma dieta saudável e alimentos regionais na modulação de fatores de risco cardiovascular. Ele comparou as diferentes recomendações dietéticas oferecidas aos pacientes e foi possível observar menor pressão arterial e glicemia de jejum nos pacientes que receberam orientações do DICABR, quando comparado a grupos que receberam orientações de diretrizes brasileiras cardiovasculares de 2012.^8^

É fundamental reconhecer que o consumo alimentar é influenciado por diversos fatores, como acesso, preferências pessoais, disponibilidade de alimentos, renda e cultura. No Brasil, a produção de alimentos é significativa, com 7,8% das terras dedicadas à agricultura e 21,2% para pastagens.^9^ Isso ressalta a necessidade de examinar as condições que afetam do início ao final desse sistema alimentar, e questionar por que a população enfrenta barreiras para acessar esses alimentos uma vez que o problema não é a quantidade produzida.

Ao formular políticas públicas e diretrizes nutricionais, é essencial considerar as complexidades do sistema alimentar brasileiro e desenvolver estratégias abrangentes que abordem as múltiplas dimensões que influenciam em todas essas etapas. Isso inclui: 1) incentivar a agricultura sustentável e a distribuição eficiente, garantindo a disponibilidade contínua de alimentos saudáveis, 2) facilitar o acesso a alimentos saudáveis através de políticas como subsídios para frutas e vegetais podem tornar esses alimentos mais acessíveis, especialmente para populações em situação de insegurança alimentar,^10^ 3) engajar profissionais de saúde devidamente capacitados para fornecer orientação nutricional individualizada aos pacientes,^11^ 4) promover mudanças nos hábitos alimentares por meio de campanhas de conscientização, educação nutricional e promoção de restaurantes com opções saudáveis, que podem auxiliar na adoção de hábitos alimentares mais adequados.^12^

Desta forma, a promoção de dietas saudáveis através de diretrizes nutricionais e políticas públicas é fundamental para combater a alta prevalência de DCV no Brasil, sendo possível identificar onde podem ser implementadas ações voltadas para estas doenças. Considerar a implementação, expansão e divulgação de programas como o DICABR, aliadas à compreensão das complexidades do sistema alimentar e à adoção de estratégias abrangentes, pode contribuir significativamente para a redução da incidência de DCV e a melhoria da qualidade de vida da população brasileira.
