# Supplementary material for: Promoção de Dietas Saudáveis para o Enfrentamento de Doenças Cardiovasculares no Brasil: A Importância das Diretrizes Nutricionais e Políticas Públicas
Source: Arq Bras Cardiol. 2024 Aug 2;121(7):e20240378. [Article in Portuguese] doi: 10.36660/abc.20240378 (PMC11341203; doi:10.36660/abc.20240378)
Supplement: Translation

# Translation: Promotion of Healthy Diets to Address Cardiovascular Diseases in Brazil: The Importance of Nutritional Guidelines and Public Policies

**DOI:** 10.36660/abc.20240378i

**Authors:** Laís Ferreira Dias, Aline Martins de Carvalho

**Affiliations:** 1 Brazil Departamento de Nutrição da Faculdade de Saúde Pública da Universidade de São Paulo, São Paulo – Brazil; 2 Brazil Instituto do Coração (InCor), Faculdade de Medicina - Universidade de São Paulo, São Paulo, SP – Brazil

**Keywords:** Cardiovascular Diseases, Food System, Disease Prevention, Public Health

Cardiovascular diseases (CVD) represent a major public health issue in Brazil, being the leading cause of mortality in the country, with coronary artery disease highlighted as the main cause of death.^1^ According to the Global Burden of Disease data, 2021, dietary patterns were responsible for 12% of the attributable risk of total deaths from chronic non-communicable diseases in the general Brazilian population.^2^ These conditions cause systemic harm, not only impacting population mortality or reducing life expectancy but also leading to disability, creating dependency for individuals to carry out their daily activities.^3^

In this context, the World Health Organization (WHO) advocates for public policies focused on health strategies and the regular updating of national dietary and nutrition guidelines, adapting to changes in dietary habits, food systems, and population health conditions.^4,5^

In Brazil, there are several nutritional guidelines and government programs aimed at promoting healthy diets. The Brazilian Cardioprotective Dietary Program (DICABR) aims to promote healthy and adequate eating to prevent heart disease, categorizing foods into groups identified by the colors of the Brazilian flag, and exploring the country’s culinary culture and regional diversity. The color green represents foods that should be consumed in larger quantities, such as fruits, vegetables, greens, milk, and yogurt. The color yellow denotes foods to be consumed in moderation, such as bread, cereals, tubers, and certain types of homemade sweets (cocada, guava paste, pumpkin sweet). The color blue represents foods that should be consumed in smaller quantities, such as meats, cheeses, eggs, butter, condensed milk, and cream. Despite its potential, adherence and dissemination among the target population were insufficient to achieve the expected results in CVD prevention.^6^

A cross-sectional study conducted with individuals at cardiovascular reference centers in Northeast Brazil revealed that the studied population is far from meeting DICABR recommendations.^7^ Their diet was low in fiber and micronutrients, with high saturated fat intake and low consumption of unsaturated fatty acids, indicating poor nutritional quality.

Another randomized controlled pilot study evaluated 117 Brazilian outpatients over 45 years old with atherothrombotic cardiovascular disease. Despite the lack of statistical significance, the study highlights the potential of consuming a healthy diet and regional foods in modulating cardiovascular risk factors. It compared different dietary recommendations given to patients, and it was observed that patients who received DICABR guidelines had lower blood pressure and fasting glucose levels compared to those who received the 2012 Brazilian cardiovascular guidelines.^8^

It is crucial to recognize that food consumption is influenced by various factors such as access, personal preferences, food availability, income, and culture. In Brazil, food production is significant, with 7.8% of the land dedicated to agriculture and 21.2% to pastures.^9^ This underscores the need to examine the conditions affecting the entire food system and question why the population faces barriers to accessing these foods, given that the issue is not the quantity produced.

When formulating public policies and nutritional guidelines, it is essential to consider the complexities of the Brazilian food system and develop comprehensive strategies that address the multiple dimensions influencing all these stages. This includes: 1) promoting sustainable agriculture and efficient distribution, ensuring the continuous availability of healthy foods, 2) facilitating access to healthy foods through policies such as subsidies for fruits and vegetables, making these foods more accessible, especially for food-insecure populations,^10^ 3) engaging properly trained health professionals to provide individualized nutritional guidance to patients,^11^ 4) promoting dietary changes through awareness campaigns, nutritional education, and promoting restaurants with healthy options, which can help in adopting more appropriate eating habits.^12^

Thus, promoting healthy diets through nutritional guidelines and public policies is crucial to combating the high prevalence of CVD in Brazil, making it possible to identify where actions can be implemented to address these diseases. Considering the implementation, expansion, and dissemination of programs like DICABR, along with understanding the complexities of the food system and adopting comprehensive strategies, can significantly contribute to reducing the incidence of CVD and improving the quality of life of the Brazilian population.
